# Computational Fluid Dynamics Methodology for Aortic Aneurysm Analysis in Computed Tomography (CT) Datasets

**DOI:** 10.7759/cureus.84523

**Published:** 2025-05-21

**Authors:** Charles G Jenkinson, Tristan L Wood

**Affiliations:** 1 Faculty of Medicine and Dentistry, Charles Sturt University, Orange, AUS; 2 Faculty of Medicine, The University of Western Australia, Perth, AUS; 3 Cardiothoracic Surgery, Prince of Wales Hospital, Sydney, AUS; 4 Cardiothoracic Surgery, Sir Charles Gairdner Hospital, Perth, AUS

**Keywords:** aortic aneurysm, computational fluid dynamics, dicom, haemodynamics, open-source software

## Abstract

Aortic aneurysms present significant clinical challenges due to the risk of rupture associated with the abnormal dilation of the aorta. Computational fluid dynamics (CFD) analysis is an emerging, non-invasive method to analyse haemodynamic forces within aneurysmal regions.

We present a detailed, reproducible workflow for the CFD analysis of aortic aneurysms based on cardiac-gated computed tomography (CT) data. Using a structured toolchain of open-source software, namely, Horos (Horos Project, Annapolis, MD, USA) for image preparation, Image Tool Kit-SNAP (ITK-SNAP) (University of Pennsylvania, Philadelphia, PA, USA) for segmentation, MeshLab (Istituto di Scienza e Tecnologie dell'Informazione-Consiglio Nazionale delle Ricerche (ISTI-CNR), Pisa, Italy) for mesh refinement, Blender (Blender Foundation, Amsterdam, Netherlands, https://www.blender.org) for boundary patching, OpenFOAM (OpenFOAM Foundation, London, UK) for CFD simulation, ParaView (Kitware, Inc., Clifton Park, NY, USA) for visualisation, and R (R Foundation for Statistical Computing, Vienna, Austria, https://www.R-project.org/) for statistical analysis, the methodology achieves high fidelity in modeling patient-specific flow conditions.

Key stages of the workflow address segmentation accuracy, mesh quality, and boundary condition assignment, ensuring that the model captures physiological flow characteristics. This approach provides a valuable and accessible tool for clinicians and researchers, supporting assessments of haemodynamic risk factors in cardiovascular research.

Our model aims to provide insights into wall shear stress (WSS), pressure distributions, and flow dynamics that may contribute to aneurysm progression and high-risk features.

## Introduction

Acute aortic syndromes are responsible for an increasing rate of deaths globally (2.79 per 100,000 person-years) [1]. Our current methods for considering surgical intervention largely revolve around absolute dimensions of the aorta and are occasionally indexed to anthropometric measurements and do not take into account blood rheology. 

We have developed a simple, reproducible, accurate, and accessible model of the ascending aorta from readily available computed tomography (CT) datasets. Although the ascending aorta is our main focus, data on the aortic arch and thoracic descending aorta are also derived. The model is then capable of undergoing analysis by a computational fluid dynamics (CFD) software package. Quantitative (wall shear stress (WSS), pressure) and qualitative (blood flow pattern) analysis of these models is performed by a steady-state solver, which models flow in an aorta assuming constant conditions.

The entire toolchain uses free and open-source software (FOSS) to further reduce the barriers for others to reproduce, verify, and improve upon our work [2,3]. Our workflow produces results similar to the work of others and could be developed into cost-effective, non-invasive clinical techniques to provide better risk stratification in aortic aneurysms [4-10].

WSS is the tangential force experienced by the endothelial surface of blood vessels caused by viscous drag from blood and may impact endothelial cellular response, potentially leading to vascular dilation and atherosclerotic disease [7].

All scripts and code to run these simulations are available on the online GitHub repository, accessible here: https://github.com/charlesjenkinson/cfd

## Technical report

Methods

**Figure 1:**
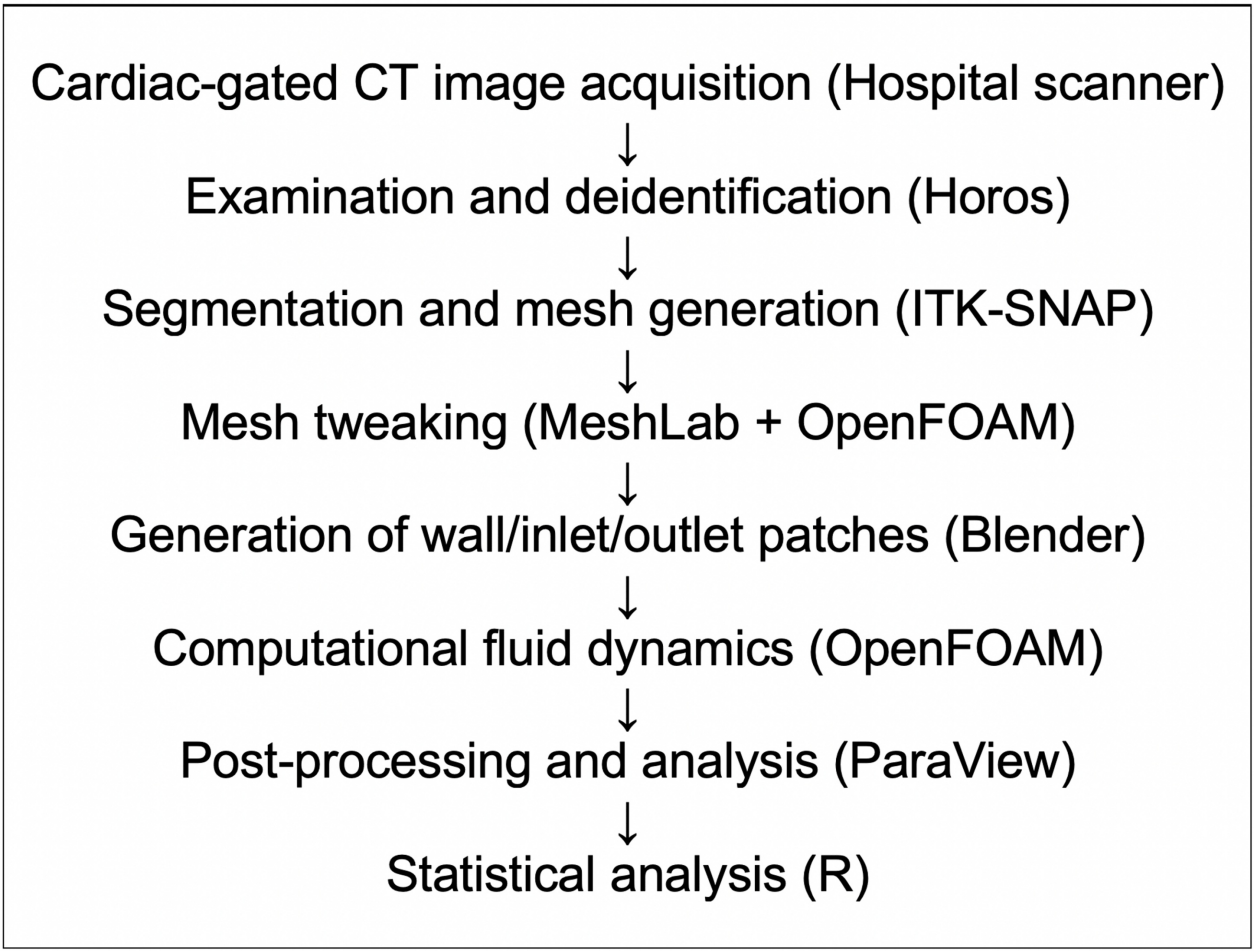
Our workflow for performing computational fluid dynamics assessment of the aorta using a FOSS toolchain. FOSS: free and open-source software; CT: computed tomography; ITK-SNAP: Image Tool Kit-SNAP

The CFD workflow includes eight main stages, each using FOSS for image processing, segmentation, mesh generation, boundary condition setup, CFD simulation, post-processing, and statistical analysis. This is demonstrated in Figure 1.

This software toolchain runs on Ubuntu 24.04 LTS (Canonical Ltd., London, UK, 2024), a FOSS operating system except for Horos (version 3.3.6, Horos Project, Annapolis, MD, USA, 2024), which is run on macOS 15 Sequoia (version 15.4.1, Apple Inc., Cupertino, CA, USA, 2025). The Ubuntu version of the Linux operating system does not have a monetary cost and can be installed on most consumer and laboratory computer hardware.

Literature review

We conducted a literature search relating to CFD of the ascending aorta using MEDLINE, Embase, and Google Scholar using the keywords "computational fluid dynamics" AND "ascending aorta". Several papers relating to CFD studies of the aorta, especially from CT images, were identified from peer-reviewed literature. We also located a master's thesis involving the use of OpenFOAM (OpenFOAM Foundation, London, UK) in a sample of three aortas [4]. Only one of these was derived from actual CT data, whereas the other two were simplified models for the purposes of developing the initial and boundary conditions. A doctorate thesis [5] involving eight healthy patients with Marfan syndrome was also identified; however, it utilised the proprietary Alya software package (Barcelona Supercomputing Center, Barcelona, Spain). Kimura and colleagues modelled 12 CT-derived bicuspid aortic valves and three controls, with boundary conditions and validation determined by magnetic resonance imaging (MRI) [6]. This was the largest study seen in our literature review. Other smaller studies were identified which only analysed the aortas of small patient numbers [7-9] or the aorta outside of the ascending region [10-13].

Cardiac-gated CT image acquisition (proprietary scanning hardware and software)

Although imaging equipment may internally store data in vendor-specific proprietary manners, the distribution of imaging usually conforms to the Digital Imaging and Communications in Medicine (DICOM) format. This standard is specified by the International Organization for Standardization (ISO 12052) [14]. It includes metadata, a series of information including patient details and demographics, details of the study, and details of the center undertaking the study.

We use the IMPAX (version 6.7.0.6011, AGFA Healthcare, Mortsel, Belgium, 2020) viewer to retrieve industry-standard DICOM files from our institution's radiology department's DICOM server. CT images were derived from multiple hospital and private radiology practice scanners.

Examination and deidentification (Horos)

CFD analysis requires good-quality DICOM images. The Horos software package is ideal to assess the suitability of imaging. Identifying metadata can be stripped from the image for purposes of confidentiality if required.

Ideal images must be acquired as arterial phase contrast-enhanced images acquired with electrocardiogram (ECG) gating in diastole from the root of the neck to at least the diaphragm, should have a slice size of 0.625 mm or smaller (though 0.5 mm is preferred), and should have minimal contrast in the venous structures (especially the brachiocephalic vein). There should be no stair-step artefact or aortic dissection. 

Segmentation and mesh generation (Image Tool Kit-SNAP (ITK-SNAP))

DICOM folders from the previous step are next loaded into ITK-SNAP (version 4.2.2, University of Pennsylvania, Philadelphia, PA, USA, 2024). Semi-automatic segmentation is carried out in "active segmentation mode". Firstly, a region of interest (ROI) is specified in all three spatial dimensions, including the entire thoracic aorta and proximal portions of the arch vessels. 

**Figure 2:**
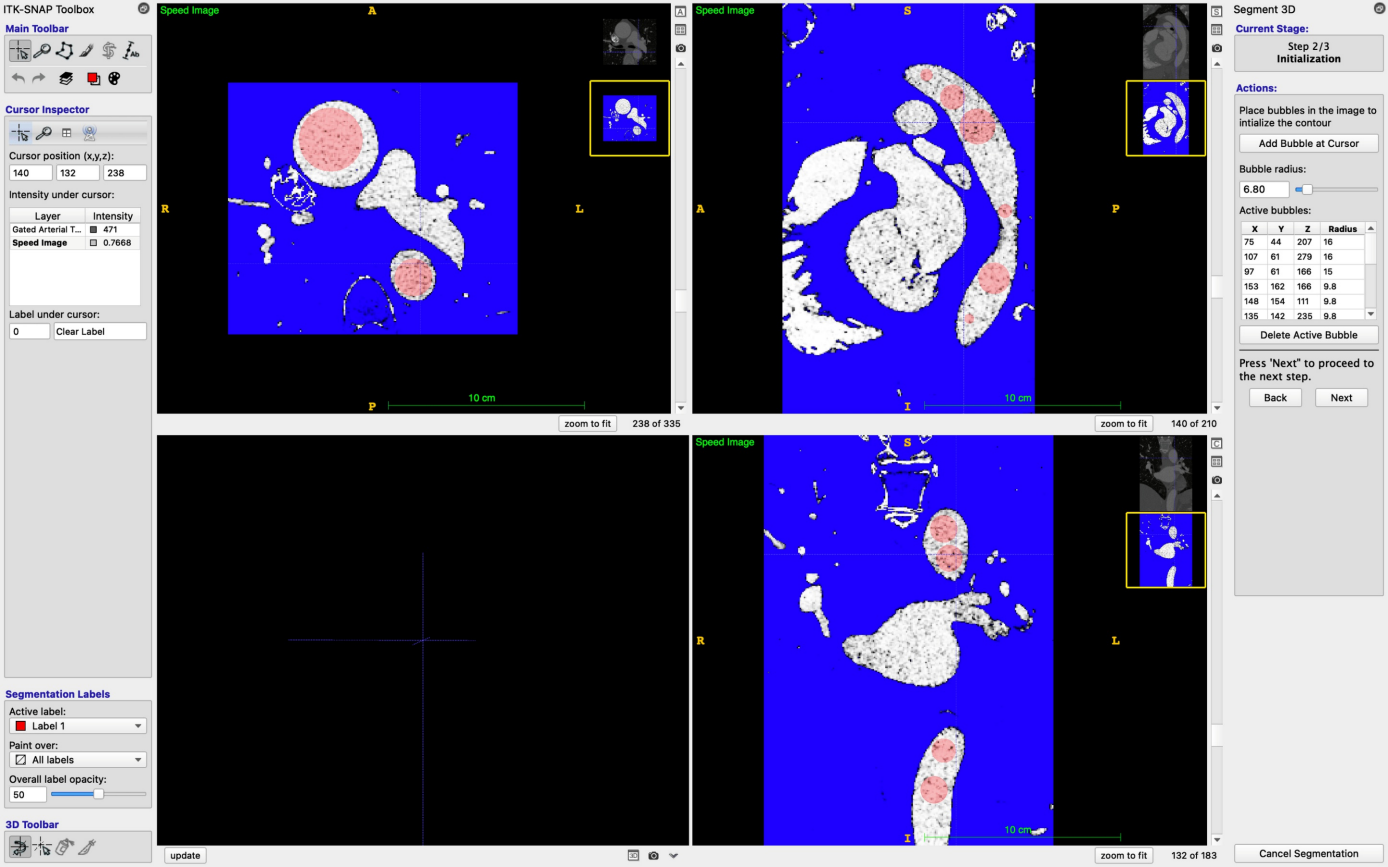
Selecting bounds in ITK-SNAP to include the entire aorta, selecting HU thresholds (note that the aorta is depicted as white), and placing bubbles in multiple planes. ITK-SNAP: Image Tool Kit-SNAP; HU: Hounsfield units

A rough mask is created by specifying a range of Hounsfield units (HU, a measure of radiodensity on CT imaging) that maximises the inclusion of the aorta, but minimises other structures such as vertebral bodies and venous structures, especially the brachiocephalic vein (Figure 2). Typical lower thresholds are around 300 HU and upper thresholds around 900 HU; however, these thresholds vary depending on contrast density within the aorta and the presence of contrast within other vascular structures.

**Figure 3:**
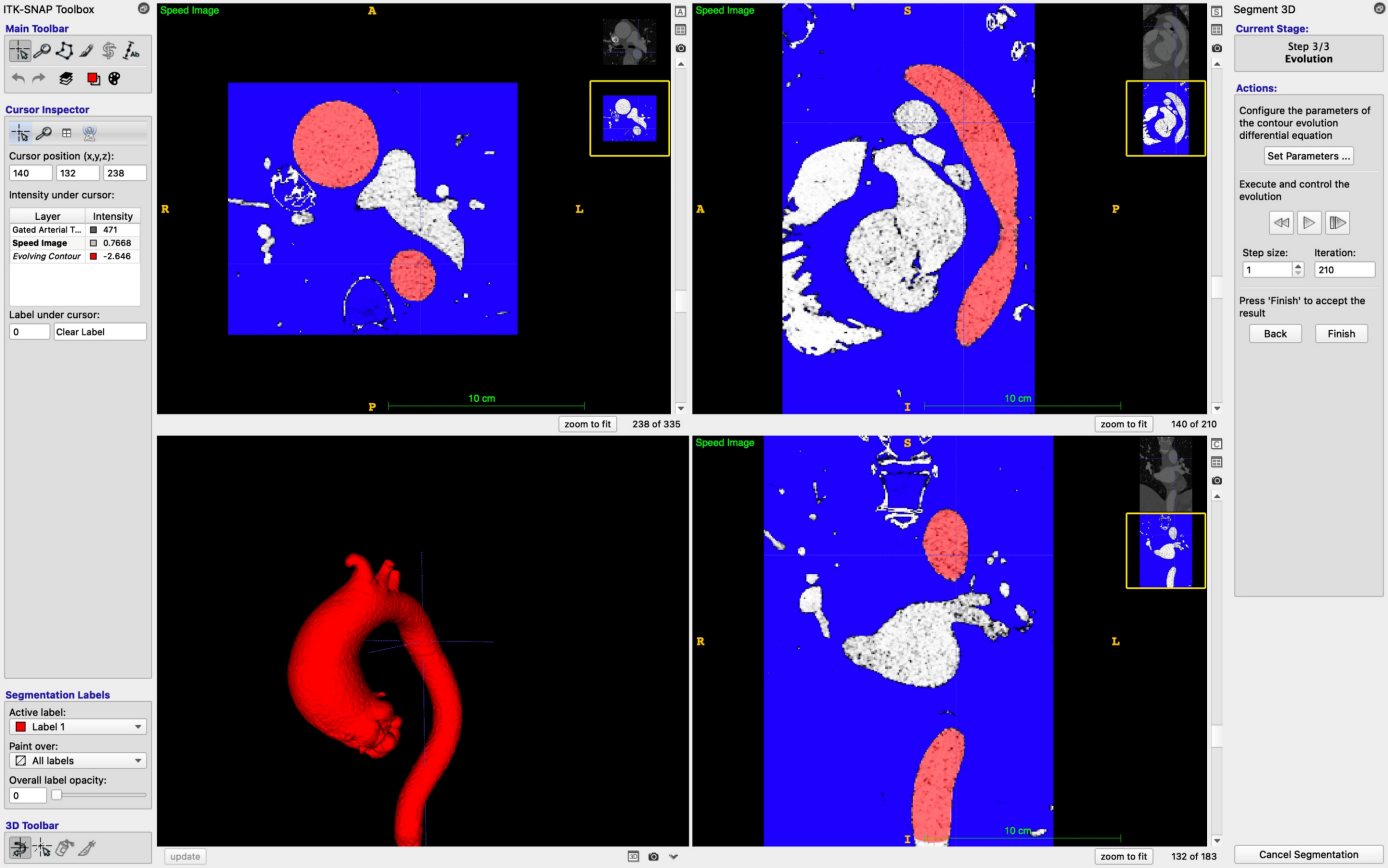
ITK-SNAP following active segment contouring process. The aorta has been segmented after the bubbles have expanded to fill the areas of similar HU (where contrast is present) and retracted from the areas of high contrast. The segmented aorta can be closely examined for accuracy at this stage. ITK-SNAP: Image Tool Kit-SNAP; HU: Hounsfield units

The resulting mask is then manually "seeded" with "bubbles". These are placed at multiple levels within the lumen of the aorta, with the size of the bubble selected individually by the eye. The semi-automatic segmentation process then "evolves" these bubbles, filling adjacent areas of similar HU values [15]. A higher number of bubbles, sized to most closely fit the aortic lumen, improves the efficiency and accuracy of the segmentation process and reduces spillover into adjacent structures of similar HU density, such as vertebral bodies, contrast-filled vessels, and the left atrium. The expansion is manually ceased when the entirety of the aortic lumen has been filled and manually scrutinised for accuracy to minimise the degree of subjectivity in this approach (Figure 3).

At the conclusion of the segmentation process, the outer bounds of the aortic lumen are represented by a series, or mesh, of triangular planes which share common edges. The mesh represents the real-life outer boundaries of where blood mixed with contrast (high HU value) interfaces with the intima of the aorta (lower HU value). The resulting mesh is exported from ITK-SNAP as a stereolithography (STL) file.

ITK-SNAP stores the location of key features of the mesh in millimetres, whereas OpenFOAM requires that STL files represent the location of these features in metres. An OpenFOAM function called *surfaceTransformPoints* is run within a terminal, having selected the folder containing the STL file as the working directory. The following command is run in the following terminal: surfaceTransformPoints -scale '(0.001 0.001 0.001)' filename1.stl filename2.stl.

This function multiplies the position of each feature of the STL file named ‘*filename1.stl*’ by 0.001, the scale factor for millimetres to metres. The output is then saved into the file ‘*filename2.stl*’. 

Generation of working STL file (Blender)

The resulting STL is imported into Blender (version 4.4.3, Blender Foundation, Amsterdam, Netherlands, https://www.blender.org, 2025). Several unnecessary default objects are created, namely, "Camera", "Cube", and "Light". A short script called *FirstDeleteThese.py* is run within the "Scripting" tab of Blender to automatically delete these items (available in the GitHub repository).

The mesh of the aorta can then be selected, and the selection inverted, capturing any artefactual points not in continuity with the aortic mesh. These are deleted, leaving only the mesh. Any other artefacts can then be selected and deleted. For simplification, we also delete the coronary and intercostal arteries, as well as calcification between the left ventricular outflow tract and aortic root. This results in a non-manifold mesh (an object which is not "bound" or "watertight") which is incompatible with CFD software and will be corrected in a subsequent step.

**Figure 4:**
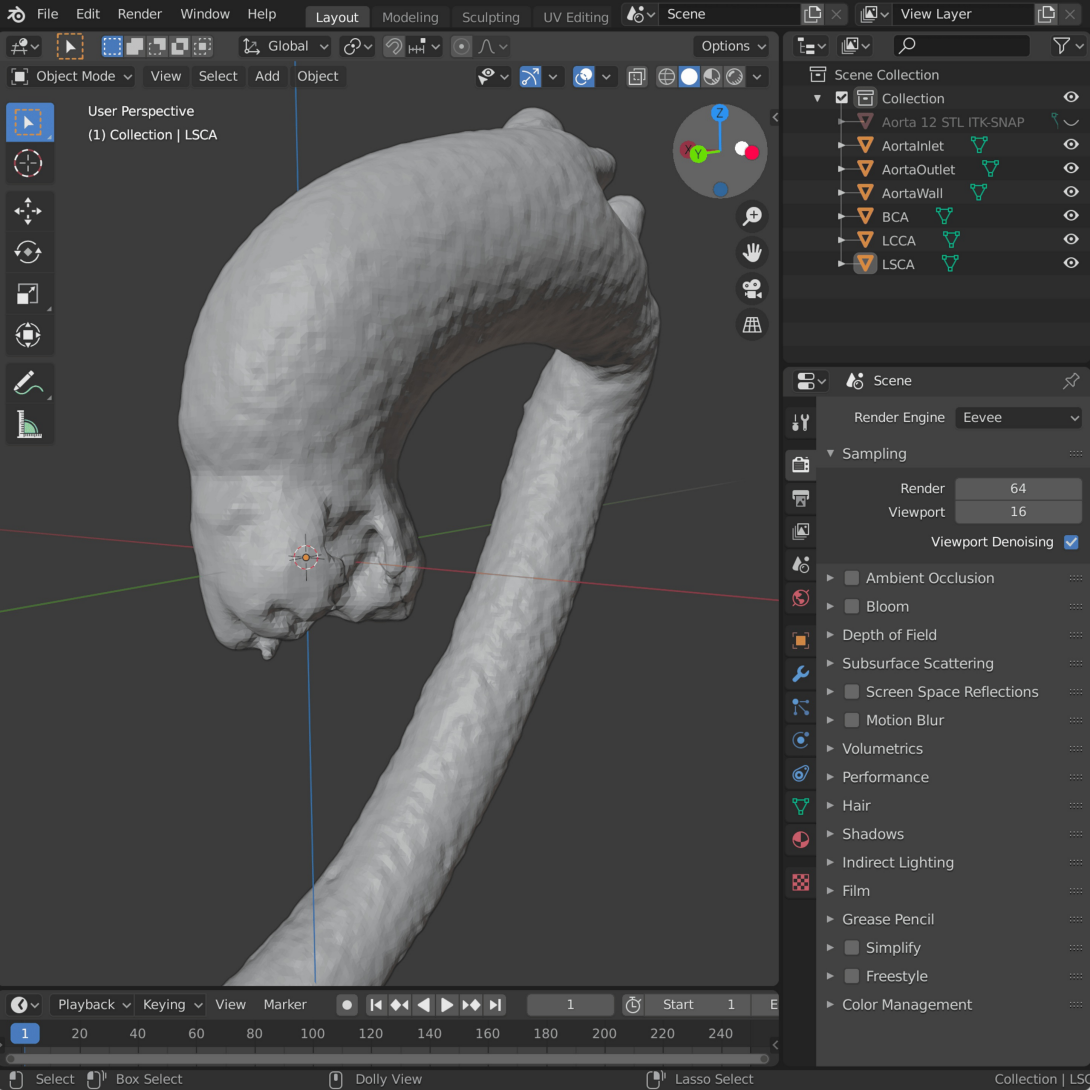
The mesh, having been processed to remove all artefact including coronary arteries and intercostal arteries, is placed in a location where the Origin (0,0,0) is bound within. The mesh is rotated until the positive Z-axis (blue, facing superiorly from an anatomical perspective) is in the simulated direction of blood flow, at 90° to the virtual basal ring, passing through the center of the sinotubular junction. LSCA: left subclavian artery; BCA: brachiocephalic artery; LCCA: left common carotid artery

The CFD analysis requires the location of a point which is specified as either inside or outside the mesh, which defines the domain to be simulated. Since this is an internal flow simulation of blood within the aorta, a fluid domain is identified located inside the closed mesh geometry. The mesh is moved until the aortic root overlies the origin (the point located at Cartesian coordinates (0,0,0)) (Figure 4). This allows a preconfigured OpenFOAM setup without having to customise the specified position of the aorta in each case.

The OpenFOAM software package (version 12, 2024) will numerically simulate fluid flow along a defined vector, originating from a smaller area of the mesh known as an inlet patch. The inlet patch specifies flow parameters (e.g., velocity, pressure) for the simulation, and the mesh must clearly define the fluid domain to distinguish between interior and exterior regions, especially important in internal flow scenarios like aortic blood flow. This requires an assumption of initial blood flow direction. We approximate this to be orthogonal to the plane of the virtual basal ring and parallel with the middle portion of the tubular ascending aorta. The mesh is rotated until the positive Z-axis (the blue axis in Figure 4) is in this direction. Standardising the initial blood flow to the positive Z-axis also allows for the reuse of OpenFOAM configuration and code with little alteration between cases.

Mesh smoothing and ensuring manifold surface (MeshLab)

**Figure 5:**
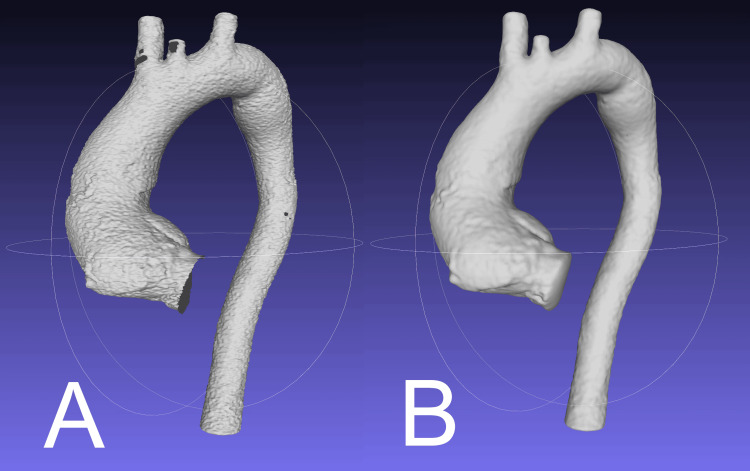
The effect of the screened Poisson surface reconstruction as before and after: the mesh in panel A has considerable surface artefact and is non-manifold (visibly discontinuous at the aortic inlet, on the brachiocephalic and left common carotid arteries, and on the descending aorta at the site of an intercostal artery). The mesh in panel B has been smoothed and no longer has these discontinuities following scaled Poisson surface reconstruction. This mesh can be divided into inlet and outlet patches for processing in OpenFOAM.

OpenFOAM requires that a mesh be manifold prior to being used. A further STL file is exported from Blender and imported into MeshLab (version 2023.12; Istituto di Scienza e Tecnologie dell'Informazione-Consiglio Nazionale delle Ricerche (ISTI-CNR), Pisa, Italy, 2023). A screened Poisson surface reconstruction is used, with the following default parameters: reconstruction depth = 8, interpolation weight = 4, minimum number of samples = 1.5, scale factor = 1.1, solver divide = 8, iso divide = 8, and smoothing iterations = 5 [16]. This ensures that the mesh is manifold and smooths out the model. A non-manifold mesh will result in an error when OpenFOAM is run; however, in our experience, the screened Poisson surface reconstruction was reliable in producing a manifold mesh. This function may correct for slight stair-step artefact and/or imperfections in the cardiac gating process (Figure 5).

An STL file containing the improved mesh is then exported from MeshLab. This file is then imported back into the Blender file for the case.

Generation of inlet/outlet patches (Blender)

Prior to CFD analysis, the mesh must be divided into inlet, outlet, and wall regions. The inlet mesh in our work is set at the level of the virtual basal ring. The primary outlet mesh is created at the descending thoracic aorta, and other minor outlet meshes are created at the extremes of the brachiocephalic artery (BCA), left common carotid artery (LCCA), and left subclavian artery (LSCA). Attention is paid to selecting the site where the vessel was "cut off" during segmentation, which corresponds to a flat plane at approximately 90 degrees to blood flow in most cases. The rheological effects of the aortic valve, coronary and thyroid ima arteries, and intercostal arteries are not considered [10-13], consistent with other models [4-8]. 

**Figure 6:**
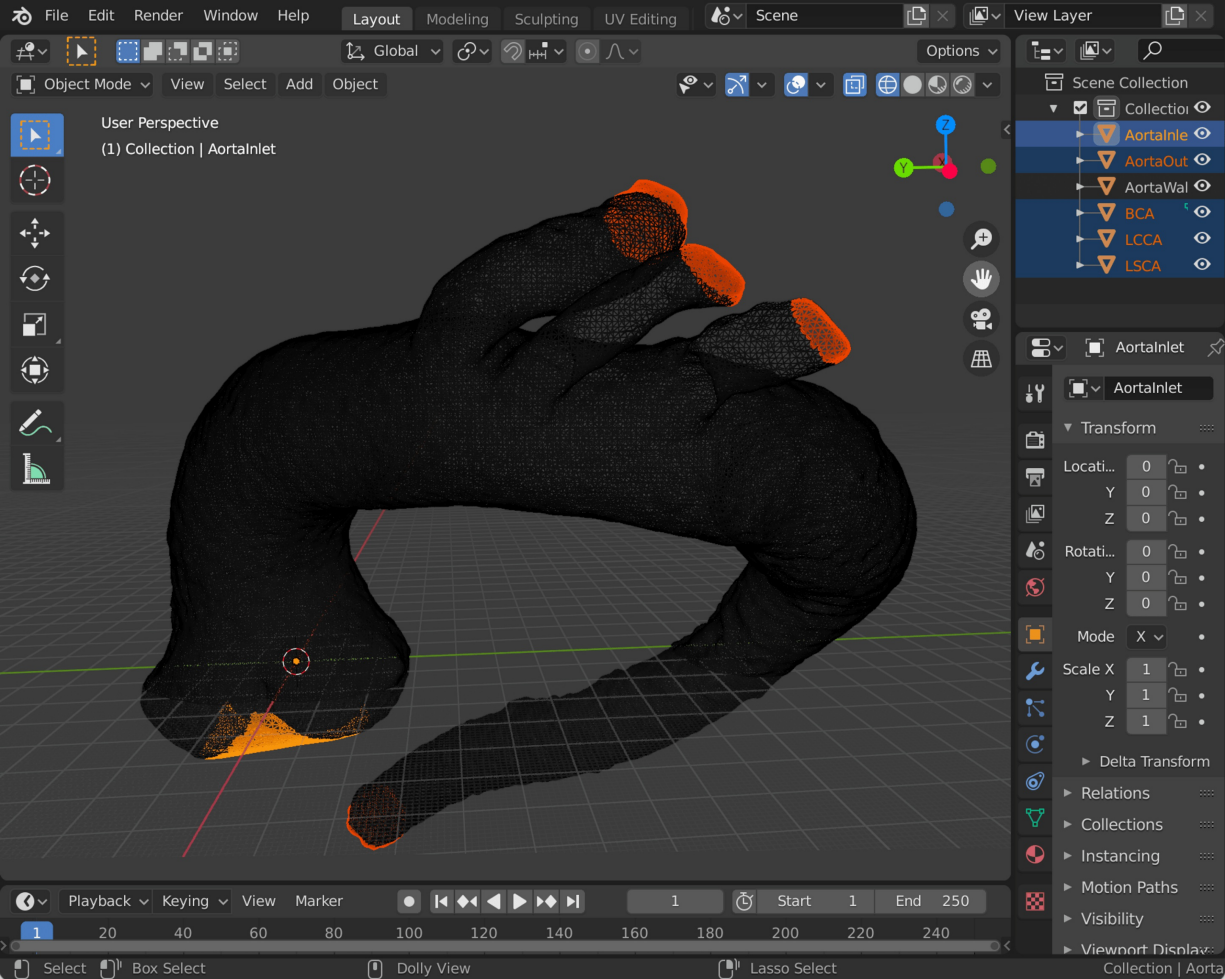
The exporting of inlet, outlet (including BCA, LCCA, and LSCA), and wall meshes. The individual inlet and outlet meshes are highlighted in this screenshot of Blender. The dark mesh remaining is the AortaWall mesh. LSCA: left subclavian artery; BCA: brachiocephalic artery; LCCA: left common carotid artery

Blender is used for the creation of inlet and outlet meshes. A circle selection tool can capture points at the inlet and outlet meshes, and these are split into a new mesh. The remaining mesh represents the wall of the aorta. As shown in Figure 6, these separate meshes are named in a standardised fashion: *AortaWall*, *AortaInlet*, *AortaOutlet*, *BCA*, *LCCA*, and *LSCA*. These are arbitrary designations we have created to standardise our mesh naming and to improve the reuse of our scripts and OpenFOAM functions across cases. To simplify the exporting process of this list of STL files, we wrote a Python script, ExportSTL.py, which can also be found in the GitHub repository. This script is run within the "Scripting" tab of Blender and exports the individual wall, inlet, and outlet meshes to a folder specified in the script, under the filename convention *AortaWall.stl*, *AortaInlet.stl*, *AortaOutlet.stl*, *BCA.stl*, *LCCA.stl*, and *LSCA.stl*. These are represented in the *OpenFOAM/system* configuration files to allow the rapid setup of cases and the reuse of code.

A shell script, *btofs.sh*, is run in the terminal to remove tags left behind by the export process in each file. This script removes "Exported from Blender-<version>" from the first line of the STL file and adds "endsolid <filename>" to the last line, to ensure compatibility with OpenFOAM.

Mesh preparation (OpenFOAM functions)

The resulting STL file is then processed by the OpenFOAM function *surfaceFeatures*, which extracts the feature edge information from the mesh file (with a feature angle threshold <150°). This is a necessary step for preparing the mesh for processing using the *snappyHexMesh* function of OpenFOAM (described later).

A background mesh of a defined size is created. This is the space in which the simulation of fluid (blood flow) will occur. This was achieved by using the OpenFOAM *blockMesh* function. The size of the background mesh was set at 1 m×1 m×1 m (creating a cube with sides each of 0.5 m distance from the origin), which captured the entire aorta in all of our simulations without clipping.

*snappyHexMesh* is then run within OpenFOAM to generate the mesh for simulation. Two bash scripts are executed in the terminal to improve efficiency: *Allrun* automatically performs each required function to create a model and run the CFD simulation without requiring operator input and contains inbuilt error checking.

After the simulation is run, *Allclean* deletes all working files and folders allowing a fresh start if the simulation faults or requires refinement. All of our scripts are included in the GitHub repository.

CFD (OpenFOAM)

The CFD simulation is then run in OpenFOAM using the *simpleFoam* solver [6]. *simpleFOAM* is a time-independent steady-state solver for incompressible, turbulent flows. Processing times will vary depending on the computer hardware. The simulation is considered complete when the differences in values of calculated quantities vary below a defined threshold between each iteration, known as a "residual" [12]. Similar to other authors [4], we used a threshold value of 1×10^-3^.

See the GitHub repository for full OpenFOAM C++ code for our model.

OpenFOAM uses a three-directory structure to set up a simulation. A summary of this is given in Table 1, with a description of each directory and file.

**Table 1:** OpenFOAM directory and file structure to set the initial conditions for the simulation. All directories and files are located within the GitHub folder *OpenFOAM*, in accordance with the structure outlined in this table. MRI: magnetic resonance imaging

Directory	Contents and function
0.orig	*/include/initialConditions* specifies the conditions that are present at the start of the simulation, including a coefficient for turbulence of blood. Values are consistent with other authors [6,13].
	*P* specifies initial pressure conditions within the aorta at time 0, which represents the start of the simulation.
	*U.orig* specifies the flow characteristics as a vector, with its origin at the inlet mesh. Several variants were tested from other authors [6,13] obtained from 4D MRI at peak systolic flow, to best approximate peak systolic conditions within the aorta.
constant	*transportProperties* sets the behavior of the fluid in the simulation to Newtonian (a valid assumption for blood flow in large vessels) and defines the viscosity of blood to 3.365×10^-6^ m^2^/s consistent with other groups [4,6,13].
	*turbulenceProperties* sets the model of turbulence for the simulation to *kOmegaSST*. This model solves for two variables: the turbulent kinetic energy (k) and specific dissipation rate (omega). It uses a k-omega formulation near the wall and switches to a k-epsilon behavior in the free stream, effectively capturing flow separation and other complex flow phenomena that are seen in the aorta, especially at bends and diameter changes.
system	*controlDict* sets the basics of the simulation. It specifies that the solver to use is *simpleFoam* and the simulation occurs over time steps from 0 to 1000 milliseconds, in 1 millisecond increments. It also informs OpenFOAM that the *wallShearStress* function is to be used.
	*snappyHexMeshDict* defines the mesh properties, including the names and details of the inlet and outlet meshes. It specifies that the layer is castellated and changes the level of detail that will be created by the *snappyHexMesh* function.

Once the OpenFOAM simulation parameters are set, the simulation is run using the *Allrun* script, followed by *Allclean*. The results for each time point are stored within the simulation folder for further analysis and visualisation in ParaView (Kitware, Inc., Clifton Park, NY, USA). The simulation is configured to record output at periodic intervals specifically, every 50 iterations, allowing only selected time steps to be written for analysis and post-processing. This is an optimisation that balances fidelity of the state throughout the simulation while reducing data size.

**Figure 7:**
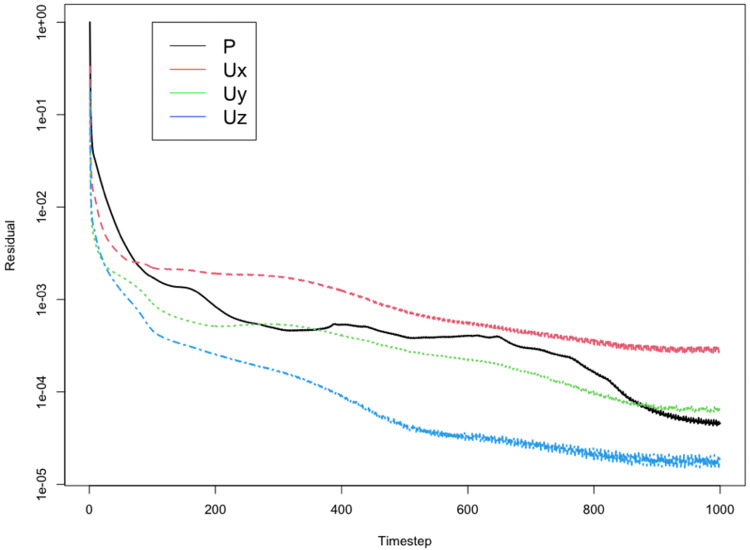
A plot of residual values over the time steps. By time step 1000, all residuals are stable and below our minimum value of 1e-03. P: pressure field; Ux, Uy, Uz: velocity components in the x (Ux), y (Uy), and z (Uz) directions

Residual values are calculated by OpenFOAM at each step of the simulation. Our target range is less than 1×10^-3^ and in our experience is consistently stable (Figure 7).

Post-processing and analysis (ParaView)

Once the simulation is complete, the solutions are viewed in ParaView (version 5.13.3, 2025). This open-source, multi-platform allows quantitative (tabulated data in .csv form) and qualitative (generation of descriptive images) data analysis. Graphical representations of blood velocity, pressure, and WSS are obtained. 

The WSS function of OpenFOAM outputs kinematic shear stress as a vector in its individual (x,y,z) components in units of m^2^/s^2^. To convert kinematic shear stress to WSS, the values are multiplied by the density of blood: ρ=1060 kg/m^3^ [6]. The magnitude of this vector is the most useful for quantitative analysis, which is determined by the equation $\sqrt{\mathrm{wallShearStress}_x^2 + \mathrm{wallShearStress}_y^2 + \mathrm{wallShearStress}_z^2} \cdot 1060$, with units in Pascals (Pa), the SI unit.

**Figure 8:**
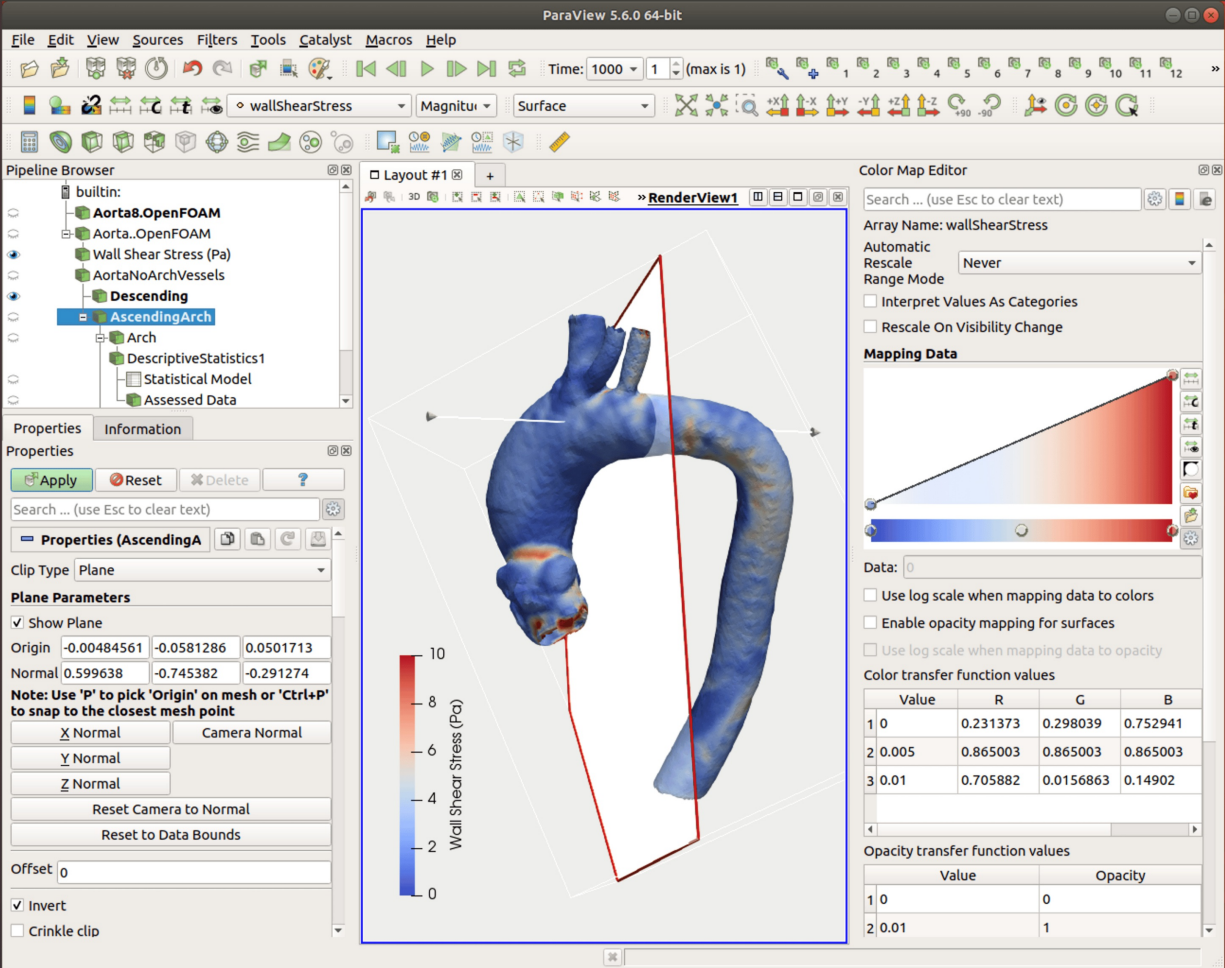
A ParaView output, depicting wall shear stress in Pascals. The aorta is broken up into ROI for quantitative analysis in anatomical regions. Note the plane (demonstrated by the red outline) between the aortic arch and descending thoracic aorta, which is defined by an Origin (not visible) and a Normal vector (the arrow with conical head and tail to indicate direction). This plane will clip off parts of the model to the left, leaving behind the descending thoracic aorta as a ROI. ROI: regions of interest

The average WSS can be determined throughout different regions of the aorta. ParaView is used to break the model up into segments using the "Clip" tool in plane mode. The aorta is divided into specific ROI based on its anatomical segments. An overview of this process is shown in Figure 8, with a detailed description in the Appendices.

The WSS values of each point of the individual regions are exported as a comma-separated value (CSV) file, another nonproprietary format. Analytical statistics can then be performed for each region of the aorta [17]. We use the R (R Foundation for Statistical Computing, Vienna, Austria, https://www.R-project.org/) software package, which is also free and open source and runs on multiple platforms. 

A plot of WSS with the aorta rotated into a left anterior oblique orientation is saved in .png format for qualitative assessment.

**Figure 9:**
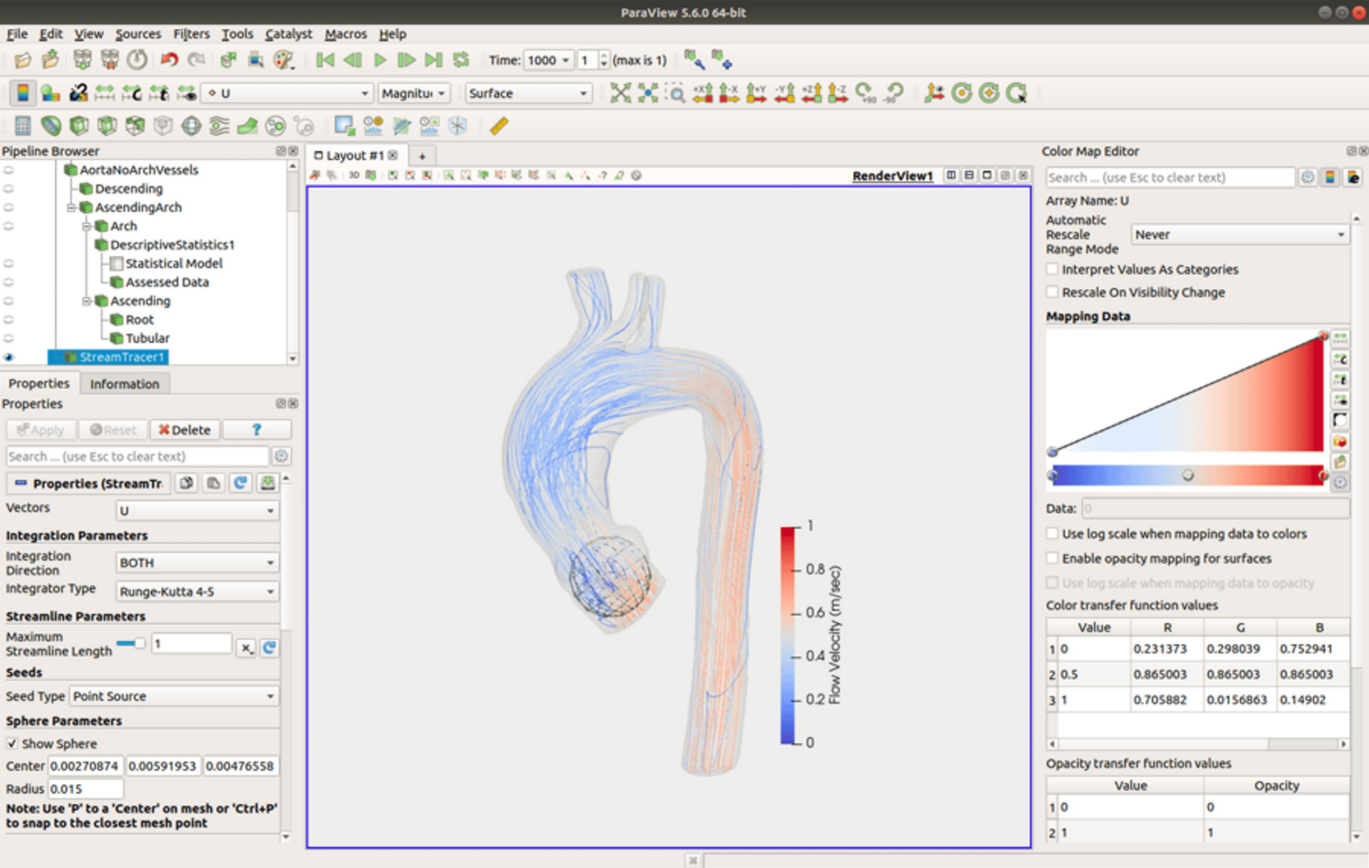
A further screen grab from ParaView, demonstrating the generation of a ribbon function. For this image, the origin "sphere" has been left visible, to demonstrate its placement within the part of the model representing the aortic root. The colour map scale demonstrates flow velocity in m/sec.

A ribbon trace is created for qualitative analysis (Figure 9). This function graphically depicts barycentric tracking, indicating the flow path and velocity, from a manually placed origin "sphere" using the Runge-Kutta 4-5 method. This was chosen as it is inbuilt into ParaView and is well validated in solvers using the Navier-Stokes equations, such as OpenFOAM [18]. We use a sphere of 2 cm placed within the aortic root, with a stream length of 1 metre, as these parameters were found to geometrically fit our models and provide sufficient coverage of the entire model length. Again, we view the model from the left anterior oblique orientation. The outline of the aortic wall is made visible with an opacity of 0.2 and coloured green (as the gradient of flow velocity varies from blue to red) which allows the magnitude of the flow velocity to be appreciated in the context of the aortic anatomy. This allows the flow patterns to be seen without the wall mesh obscuring stream line detail while still allowing anatomical context.

**Figure 10:**
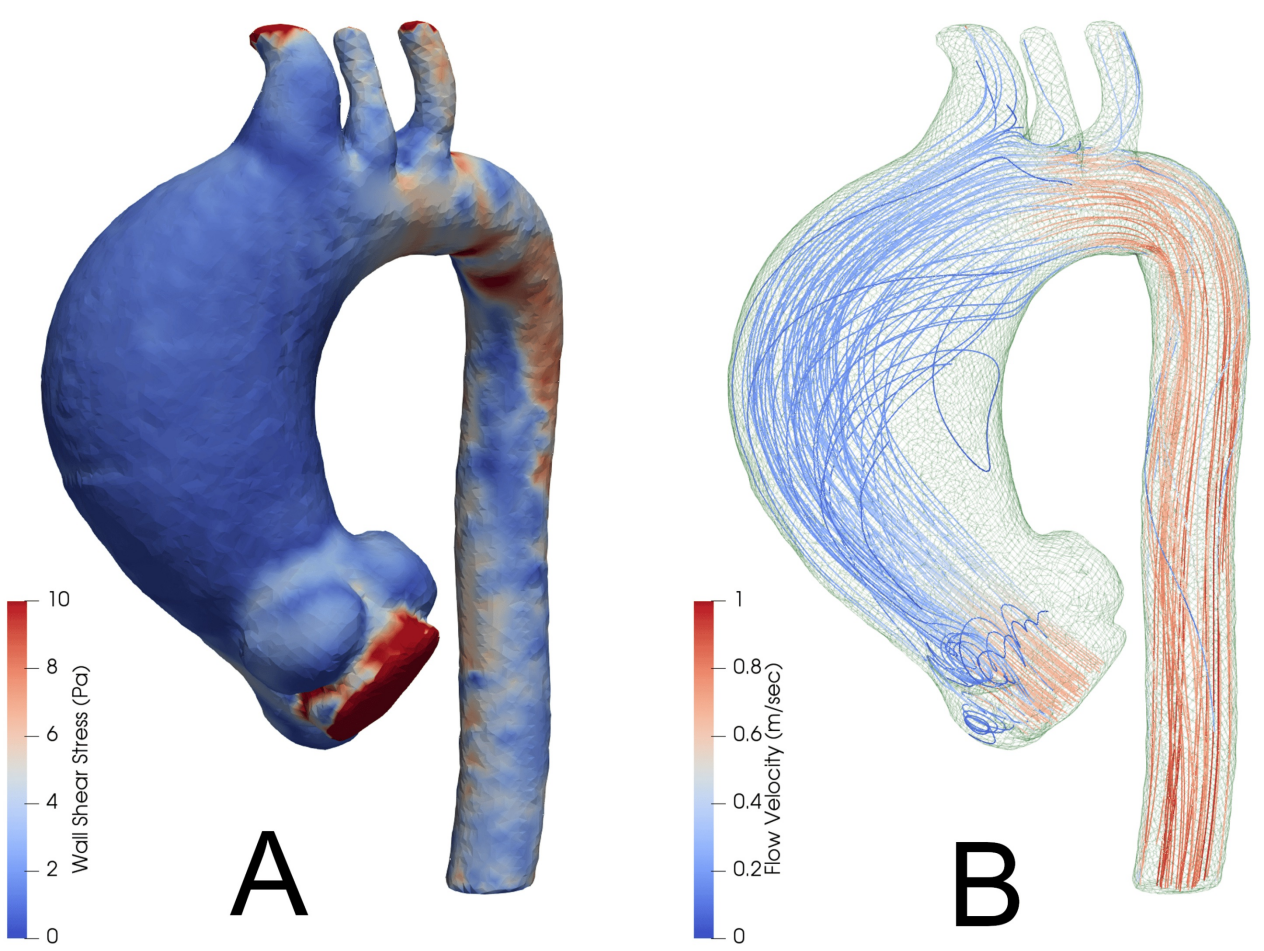
Example output from ParaView. Wall shear stress is demonstrated by panel A (measured in Pascals), and flow characteristics are demonstrated by panel B (with colour scale in m/sec).

Output images are created for both WSS and flow characteristics. An example output is shown in Figure 10. WSS and patterns of simulated blood flow are similar to other studies where CT images were also validated with MRI [6].

A .pvsm "state" file is created, which saves the location of the ROI planes, WSS calculations, and image configurations. This allows further analysis or the future use of other solvers.

## Discussion

Limitations of our model

There are some significant assumptions and limitations of this model with respect to real-world physiology. Many of these are amenable to refinements in the modelling techniques that can be employed with more advanced CFD simulation and are a subject of ongoing work.

Like any CFD model, a good-quality dataset is essential. In clinical practice, scans of necessary quality are not ubiquitous. Indeed, when we retrospectively reviewed 215 clinical scans for CFD, only 18 were technically suitable, mainly due to excessive slice thickness, poor ECG gating, and stair-step artefact. In our clinical practice, we now routinely request gated CT aortograms which has eliminated this issue.

Our simulation uses the simpleFoam solver, which approximates conditions at peak systole. This is somewhat of an oversimplification. simpleFoam solves for steady-state values, in effect simulating constant blood flow. In a pulsatile circulation, a steady-state condition is not seen. However, transient state time-dependent flow phenomena solvers require significantly more processing power, time, and storage and are more sensitive to mesh and initial conditions. This is the subject of ongoing work.

The mesh created by OpenFOAM forms a solid, incompressible boundary without distensibility or wall thickness and does not consider the effect of left ventricular dynamics on aortic flow. The modelling of wall thickness and distensibility is difficult, especially in vessels which may be diseased or contain significant atheroma [12]. They typically require specialised solvers (e.g., pimpleDyMFoam, dynamicMeshDict setup) and mesh motion or morphing strategies (e.g., arbitrary Lagrangian-Eulerian (ALE), sliding interfaces, or overset meshes). This will likely remain a drawback of CFD in cardiovascular modelling for the foreseeable future.

Our model uses standard values of initial blood flow conditions, similar to experimental data from other authors [4]. We approximate the direction of the vector of blood flow across the aortic valve, using a simplified model where the direction of blood flow is simulated in the direction of the positive Z-axis: the positive Z-axis is typically aligned normal to the inlet (positioned at the virtual basal ring), defining the direction in which the flow enters the domain. This is common in cases where the flow is assumed to be entering from a boundary at a fixed angle (usually perpendicular to the inlet surface). Gravity is not considered as a force acting on the blood; however, it is negligible relative to pressure-driven flows in models such as this. Others [6] have used superimposed datasets from 4D MRI, albeit with a far higher cost and complexity. Swanson and colleagues [9] used Doppler ultrasound in a series of three paediatric patients with aortic coarctation to measure blood velocities, and this is a good solution which we are currently investigating. 

Our model does not include the aortic valve (and, to a lesser importance, the coronary arteries). Given this is also a steady-state model, the effects of aortic regurgitation are also not considered. This has been investigated by others [6,13]. This limitation will be addressed in a future non-steady-state solver, though the effect of the coronary arteries should be negligible.

Despite these limitations, we produced a model that was capable of performing CFD calculations on appropriate CT scans, with results similar to other studies, and real-world figures. Residual values (as mentioned before) were small and within our target range of less than 1×10^-3^.

Directions for future research

Our current model of aortic aneurysm is simple, accessible, reproducible, and cost-effective. In the future, we intend to improve on our methodology in numerous ways, with the same aims in mind.

For further refinement of our model, echocardiographic and Doppler ultrasound measurements will be taken to determine true flow velocities. This would also allow better modelling of inflow direction and provide boundary conditions more in keeping with the patient's normal physiology. By measuring pulse wave Doppler, it would also be possible to model pulsatile flow through the inlet mesh of the model. There are solvers available in the OpenFOAM software package that have this capability, albeit with added complexity. This would also allow the measurement of changes in WSS over the cardiac cycle, as well as the measurement of oscillatory shear index.

Future models will include coronary arteries as well as more accurately model the aortic valve. This is especially important in bicuspid aortic valves, where the aortic inflow can be eccentric and turbulent, with complex WSS interactions in small regions of the aorta [6,19].

In the future, our model will be compared with 4D MRI, as comparing CFD simulations with 4D MRI data is a valuable approach for validating and improving the accuracy of the CT-derived models. In previous small-series validation studies [20], fluid velocity profiles and WSS agreed with those measured by 4D MRI.

With real-world validation, kinematic shear stress and WSS could help to identify patients at risk of acute aortic syndrome in a way which is cheap, reproducible, and non-invasive, in addition to aortic size parameters, as is the current norm. This is also the topic of our ongoing research.

## Conclusions

We have produced a CFD model of ascending aortic aneurysm based on a CT dataset that relies on an entirely free and open-source toolchain, using computer hardware within the reach of most researchers. This is an example of FOSS lowering barriers to medical research, as well as allowing complex work to be done. Our model does make assumptions (for instance, steady-state flow and rigid walls) to simplify the model and has clear, stated limitations. We are continuously improving the accuracy and reliability of the model while maintaining a FOSS toolchain. Our hope is that others are able to utilise this model and iterate upon it, both for the improvement of our techniques and to demonstrate the strengths of FOSS within a medical research context.

## Appendices

Further description on creating ROI in ParaView

The ParaView model can be further broken up into segments for individual analysis. This is possible by using the "Clip" tool in plane mode. This tool defines a plane specified by an origin and a vector with an orthogonal direction to this plane (otherwise known as a *normal*). This plane is used to clip the model in the direction of this normal vector, leaving behind a new ROI. By creating stepwise ROI with successive planes, the model of the aorta can be divided into anatomical segments. This was seen in Figure 6.

All planes are created as close as possible to right angles to the aorta wall mesh and positioned by anatomical definitions [1]. They are created in a stepwise fashion. The following process successively segments the aorta into the appropriate ROI. Some are used only to exclude segments of the aorta which would interfere with subsequent clipping steps and are not included within the analysis. Following these steps on an actual model should make this clear. A sample ParaView state file is included in the GitHub repository under the *Samples* directory for reader experimentation, without having to perform the entire CFD process.

A plane is created first across the aortic arch at the level of the arch vessels and clipped to retain the model of the ascending, arch, and descending thoracic aorta. This is named ‘AortaNoArchVessels’ and appears in the Pipeline Browser, as demonstrated in Figure 7. A further plane is then created at the boundary of the arch and descending aorta. The descending aorta is then isolated and named ‘Descending’. The AortaNoArchVessels ROI is then selected again, and a copy of the plane for the descending is made. To select the opposite ROI (that is, the ascending aorta and aortic arch), the direction of the normal vector is reversed by multiplying the numerical values defining the vector by -1: in the case of the plane in Figure 8, this would yield the result (-0.599638, 0.745382, 0.21274). (The numerical values of the Origin point and the Normal vector are visible under the Properties tab in the screenshot.) The result of this operation is named ‘AscendingArch’. A further plane is then generated at the level of the transition from the ascending aorta to the arch, leaving behind the arch. This is named ‘Arch’. This plane is inverted again by multiplying the numerical values of the Normal vector by -1 and applied to the AscendingArch: the result is named ‘Ascending’. A final plane is created at the sinotubular junction of the model, which leaves the ‘Tubular’ ROI. The Normal vector is again multiplied by -1, to leave the ‘Root’ ROI. This process allows the complete division of our aortic model into separate anatomical segments, which may be quantitatively assessed individually.
